# Development of a Multi-Array Pressure Sensor Module for Radial Artery Pulse Wave Measurement

**DOI:** 10.3390/s20010033

**Published:** 2019-12-19

**Authors:** Donggeun Roh, Sangjin Han, Junyung Park, Hangsik Shin

**Affiliations:** Department of Biomedical Engineering, Chonnam National University, 50 Daehak-ro, Yeosu 59626, Korea; nodg0426@gmail.com (D.R.); tkdwls9045@gmail.com (S.H.); junyung.pak@gmail.com (J.P.)

**Keywords:** blood pressure, multi-array pressure sensor, pulse wave measurement, radial artery

## Abstract

This study proposes a new structure for a pressure sensor module that can reduce errors caused by measurement position and direction in noninvasive radial artery pulse wave measurement, which is used for physiological monitoring. We have proposed a structure for a multi-array pressure sensor with a hexagonal arrangement and polydimethylsiloxane that easily fits to the structure of the radial artery, and evaluated the characteristics and pulse wave measurement of the developed sensor by finite element method simulation, a push–pull gauge test, and an actual pulse wave measurement experiment. The developed sensor has a measuring area of 17.6 × 17.6 mm^2^ and a modular structure with the analog front end embedded on the printed circuit board. The finite element method simulation shows that the developed sensor responds linearly to external pressure. According to the push–pull gauge test results for each channel, there were differences between the channels caused by the unit sensor characteristics and fabrication process. However, the correction formula can minimize the differences and ensure the linearity, and root-mean-squared error is 0.267 kPa in calibrated output. Although additional experiments and considerations on inter-individual differences are required, the results suggested that the proposed multiarray sensor could be used as a radial arterial pulse wave sensor.

## 1. Introduction

Blood pressure is one of the important human vital signs and is an important parameter that is always monitored and managed in the clinical setting. Blood pressure is defined as the pressure with which blood pushes against the blood vessel wall; it changes with the inflow and outflow of the heart. The blood pressure value changes between the lowest value in ventricular relaxation and the maximum value in ventricular contraction, based on the mean intra-arterial pressure. In a clinical setting, blood pressure is generally measured and used as a representative value, such as systolic blood pressure, diastolic blood pressure, mean arterial pressure, and pulse pressure [1]. Since blood pressure is a continuous value in nature, such blood pressure readings may be referred to as intermittent blood pressure. In order to measure the intermittent blood pressure, a method based on sound or vibration caused by the turbulence generated by occlusion or flow, the blood of the artery is mainly used. Blood pressure has been measured directly and indirectly. To measure blood pressure directly, an invasive catheter embedding a pressure sensor on the tip is generally used through intra-arterial cannulation. However, invasive measurement methods are cumbersome and burdensome for the patient. In addition, there is a limitation they cannot be used in daily life, therefore, indirect blood pressure measurements such as auscultation or oscillometry have been popularized. Auscultation is a method of estimating blood pressure based on sound, while the method of estimating blood pressure based on vibration is called oscillometry [2]. The auscultation method estimates systolic and diastolic blood pressure based on Korotkoff sounds, while the oscillometry method estimates systolic and diastolic blood pressure using a proprietary formula, measuring the mean arterial pressure at the point where the fluctuation of pressure in the cuff caused by the pulsation is maximized [3,4]. This method has the advantage of being non-invasive, but it requires at least a few minutes to re-measure, so there is a limit in that one cannot measure rapid change of blood pressure. Continuous blood pressure is known to provide hemodynamic information beyond simply maximum and minimum blood pressure values [5,6,7]. In fact, the blood pressure waveform is known to provide vascular stiffness and hemodynamic information, and can also be used to calculate an augmentation index related to cardiovascular disease risk and aging of the arteries [8,9]. Continuous blood pressure is a key indicator that should be measured in patients who need intensive health care [10]. However, because of the burden of invasive measurement methods, continuous blood pressure is measured only for patients who cannot avoid measurement, such as anesthetized, surgical, or critical patients. As a method for non-invasive measurement of continuous blood pressure and hemodynamic information, volume clamp methods, such as the Finometer^TM^ (FMS, Finapres Measurement Systems, Arnhem, The Netherlands) and arterial tonometry, have been proposed [11,12]. Among these, arterial tonometry directly measures the change in blood pressure in the radial artery by using a pressure-measuring probe on the skin. Tonometry is known to measure continuous blood pressure non-invasively. However, it is rarely used as a standard clinical method, because it requires a skilled operator who can accurately locate the artery and compress it to an appropriate level [13,14].

To measure a pulse wave on the radial artery, previous studies suggested a multi-channel pressure sensor arranged in a grid to accurately position the sensor in the center of the radial artery [15,16]. However, since the individual pressure sensors that make up the multi-channel pressure sensor must be present on the radial artery, when designing the multi-channel pressure sensor, the dimensions and arrangement of the individual sensors should be considered first. In addition, when the pressure is applied, the aspect of the pressure distribution depends on the sensor arrangement, so a design that minimizes the pressure variation applied along the direction of the sensor is required. In this study, we proposed a new multi-channel pressure sensor to improve the accuracy of radial artery pulse wave measurement. This paper explains the design of a sensor structure in which several pressure sensors of about the same size as the radial artery are arranged in a hexagonal shape, and provides a characteristic test result of a sensor using a force-measuring device and an actual pulse wave measurement result. The findings provide information on the development and verification of new sensors.

## 2. Materials and Methods

### 2.1. Development of Multi-Array Pressure Sensor Module

**Figure 1:**
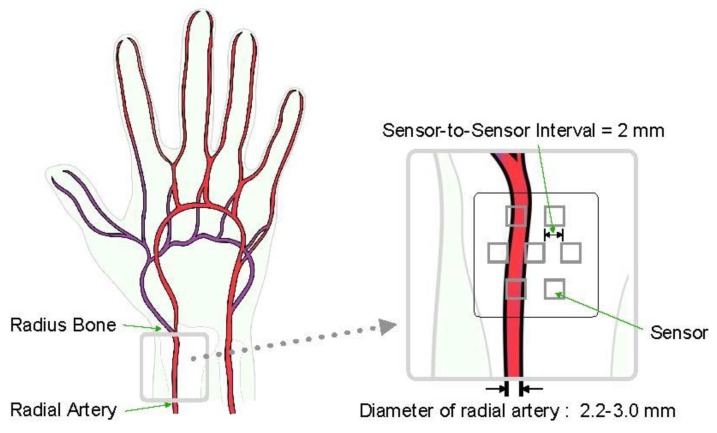
Sensor concept design based on anatomy of radial artery.

In this study, we developed a multi-channel pressure sensor module that has hexagonal unit sensor placement to minimize the effects of wearing position and directional deviations of sensors for radial artery pulse wave measurement. The sensor module has a structure in which piezo-resistive pressure sensors are staggered. When designing the sensor module, it was difficult to place the sensor in the correct artery position on the skin, so we focused on placing the sensors in such a way that at least one sensor could be placed on the artery. In sensor placement, the mean diameter of the arteries is generally known to be about 2.2–3.0 mm [17,18,19,20,21,22,23], so the distance between the sensors is minimized and finally set to 2.0 mm by the specification and attachment method of the sensor (see Figure 1).

**Figure 2:**
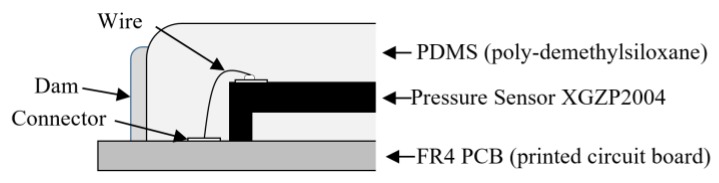
Sensor concept design based on anatomy of radial artery.

As a result, two, three, and two sensors were placed in the three rows. A piezo-resistive pressure sensor unit, XGZP2004 (CFSensor Co., Ltd., Wuhu, China) [24], which is composed of a springy diaphragm and four resistors integrated in the diaphragm, was used as a unit pressure sensor constituting the multi-array pressure sensor (see Table 1). According to the manufacturer’s manual, in each sensor unit, “Four piezo-resistors form a Wheatstone bridge structure. When the springy diaphragm is pressured, Wheatstone bridge produces a linear millivolt voltage that is proportional to input pressure” [24]. The sensor was connected to the PCB using an electrical wire, as represented in Figure 2, coated with 1.27-mm-thick polydimethysiloxane (PDMS) to protect the wire. We used Sylgard-184 (Dow Corning, Midland, MI, USA) as the PDMS. The dam structure was designed around the sensor mounting surface to coat the PDMS at a predetermined position and prevent run off. Dams were fabricated in a 17.6 × 17.6 × 0.74 mm (width × height × thickness) rectangular region with a width of 1 mm using KE-45 (Shin-Etsu Chemical Co., Ltd., Japan), a silicone material. Finally, the sensor module was designed with seven individual sensors arranged in a hexagon measuring 17.6 × 17.6 mm (width × length).

**Figure 3:**
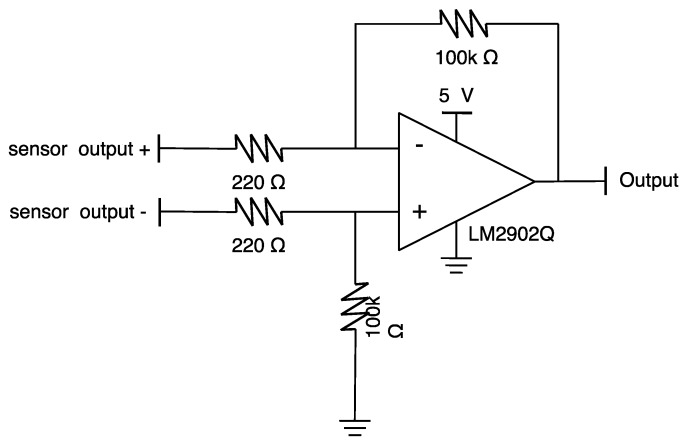
An amplifier for a channel of the pressure sensor.

**Figure 4:**
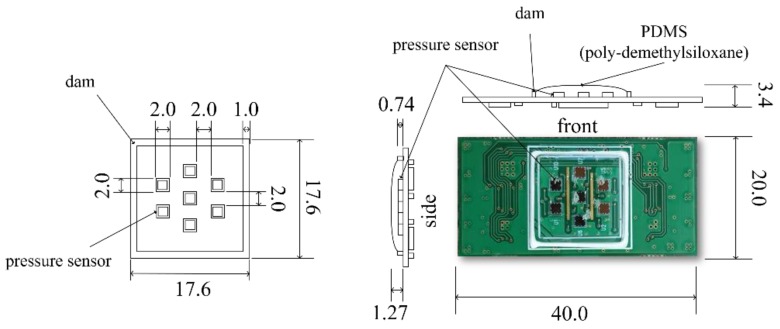
Design of the multi-array pressure sensor module.

Since the pressure sensor used has a maximum differential voltage output between 50 and 90 mV, when using an analog–digital converter with an input range of several V, signal amplification is required. In order to amplify the signal generated by each pressure sensor, the analog front end, combining the amplification circuit shown in Figure 3 for each channel, is configured on the back of the multi-channel pressure sensor module. The gain of the amplifier circuit was set to about 455. Finally, the size of the fabricated multi-channel pressure sensor module was 40.0 × 20.0 × 3.4 mm (length × width × thickness), as shown in Figure 4. PDMS thickness was determined as 1.2–1.3 mm empirically to protect wire-bonding and to minimize the attenuation of sensitivity. The PDMS thickness of 1.27 mm in Figure 4 shows the actual measured value of the fabricated sensor.

### 2.2. Finite Element Method Modeling for Sensor Evaluation

**Figure 5:**
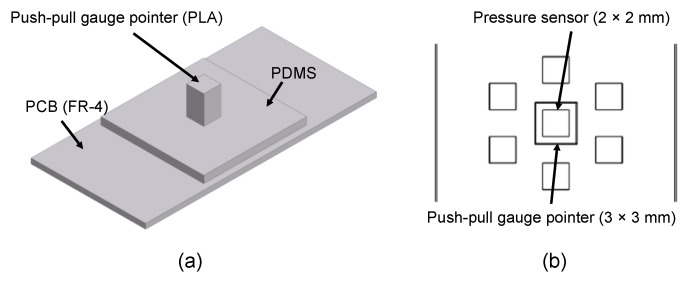
Three-dimensional model and dimensions for the finite element method analysis: (**a**) side view and (**b**) top view of the jig sensor supporter structure.

We used finite element model (FEM) analysis to decide how the pressure would be delivered to the sensor from the pressurization unit. We performed the FEM analysis using MeshFree 4.0 (MIDAS IT Co., Seongnam, Republic of Korea), a numerical analysis program using an automatic alignment grid technique. To simulate the same conditions as in the actual pressure sensor pressurization test, we created a 3D model to pressurize the pressure sensor in one of the multi-channels using a pointer-type jig, as shown in Figure 5. In the generated 3D model, the pressure sensor was created with the same structure and size as for the actual pressure sensor; the jig pointer was 3 × 3 × 5 mm (width × height × thickness). In the simulation, linear static analysis was applied to observe the stresses in the pressure sensor caused by the contact between the PDMS and the pressure sensor when the multi-channel pressure sensor was pressurized. Load conditions were set at 0.05 N intervals from 0.05 N to 1.3 N on the top of the pointer jig. In addition, the contact conditions were set to the underside of the pressure sensor supporter, and each contact condition of the generated shape was set to the Weld condition. The material specifications, such as elastic modulus and Poisson’s ratio of a single pressure sensor, for the PCB, PDMS, 3D pressure sensor supporter, and jig that make up the multi-channel pressure sensor, are shown in Table 2.

### 2.3. Pressure Test Using a Pointer Jig

**Figure 6:**
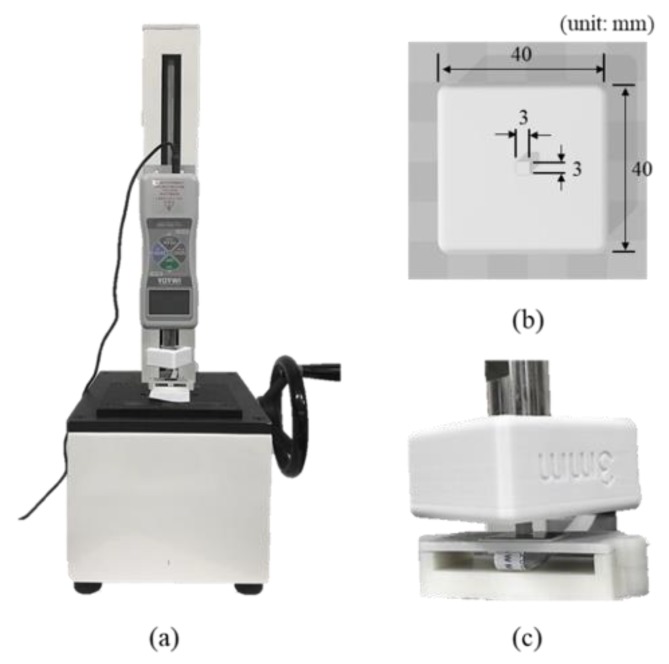
Push–pull gauge system and pointer jig for pressure test: (**a**) push–pull gauge setup, (**b**) pointer jig and (**c**) pressure test using pointer jig.

In order to quantify the degree of attenuation of the force delivered from the PDMS to the pressure sensor when the externally coated multi-channel pressure sensor with PDMS was pressurized, we did the pressure test on all the channel pressure sensors constituting the multi-channel pressure sensor. For each of the single pressure sensors, we obtained data by manually increasing the value from 0 N (before pressurization) to the force at which the sensor output became saturated. At this time, the increase in pressure was adjusted to have two measured values within 0.1 N intervals; the pressure was increased manually and repeated three times in total. A DS2-5Npush–pull gauge (Imada Co., Toyohashi, Japan) measured the force used to press the sensor and a push–pull gauge stand was used to finely adjust the force applied to the sensor. In order to pressurize a single pressure sensor, we used a jig measuring 3 × 3 mm (width × length) of the contact pointer area. Figure 6 shows the shape and size of the measuring tool and jig used.

### 2.4. Pulse Wave Measurement Test

**Figure 7:**
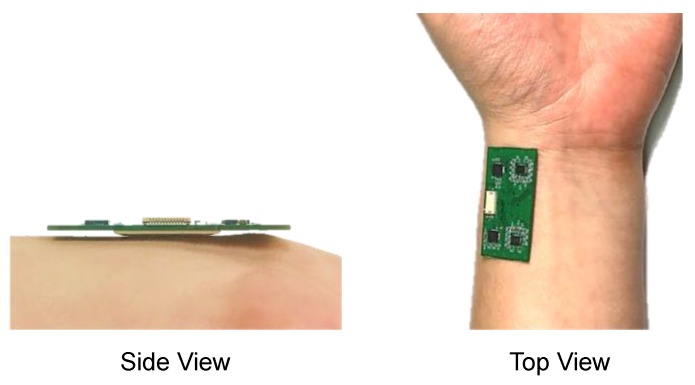
Sensor positioning for radial artery pulse wave measurement.

In order to confirm the feasibility of the radial artery pulse wave measurement of the developed multi-channel pressure sensor module, the sensor module was placed near the left radial artery and pressed with the opposite hand to check whether the pulse wave was measured. Signals were measured in a healthy adult male in his twenties. The sensor unit was in contact with the position where the pulsation was felt. Signals were recorded at a 1 kHz sampling rate for each channel for a total of 10 s using a separately developed in-house analog–digital conversion system and computer application for pulse wave measurement. The signal was measured by touching the developed sensor on the wrist, as shown in Figure 7.

## 3. Results

### 3.1. FEM Analysis Result

**Figure 8:**
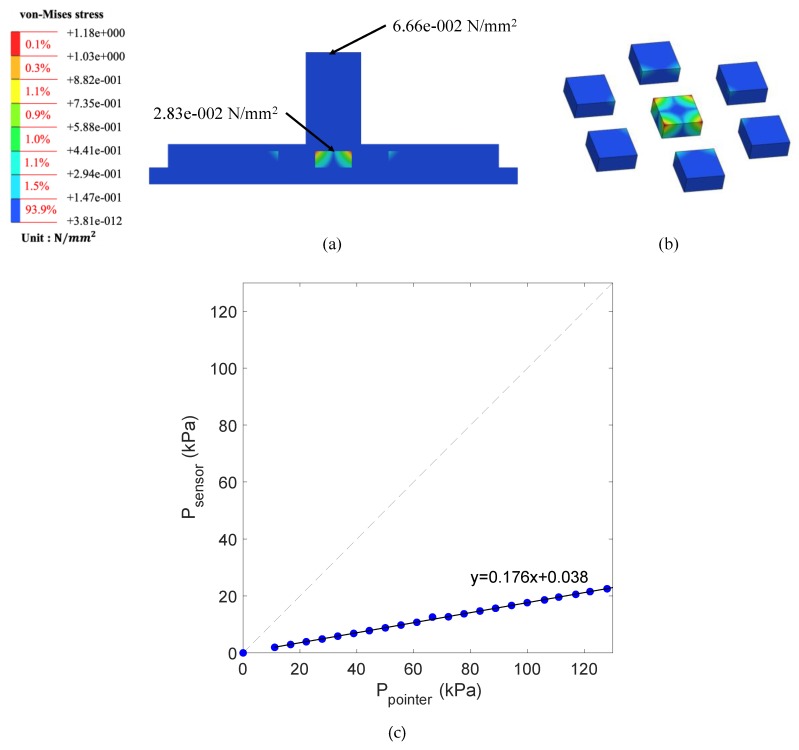
Result of FEM analysis: (**a**) FEM simulation results for jig-sensor supporter; (**b**) pressure distribution for each channel when pressure is applied to the center channel (pressure values are exaggerated to emphasize the distribution); (**c**) simulation result. The slope of the regression equation between the force applied through the jig pointer and the force on the sensor surface is 0.176.

Figure 8 shows results of FEM simulation and the von-Mises stress distribution delivered to the sensor and sensor support when pressurized with a force of 0.6 N in the center of the multi-channel pressure sensor. From the simulation results, the pressure applied to a point on the PDMS contact can be attenuated to approximately one-fifth when delivered to the pressure sensor covered by PDMS. For example, if a force of 0.6 N is applied to a jig measuring 3 × 3 mm^2^, a force of 6.66 × 10^−2^ N is applied to the unit area. In this case, FEM simulation shows that a force of 2.83 × 10^−2^ N was applied to the surface of the sensor chip, and a total of 0.113 N was applied to the 2 × 2 mm^2^ area. From this result, it can be seen that the 0.6 N pressure applied outside the PDMS is transferred to the pressure sensor chip at 0.113 N, which is attenuated by approximately 81.2%, equivalent to 14 dB damping. When the center of the multi-channel pressure sensor is pressed through the pointer jig, it can be seen that the greatest stress is applied to the sensor placed in the pressurized position among the sensors.

### 3.2. Pressure Test and Calibration Results

**Figure 9:**
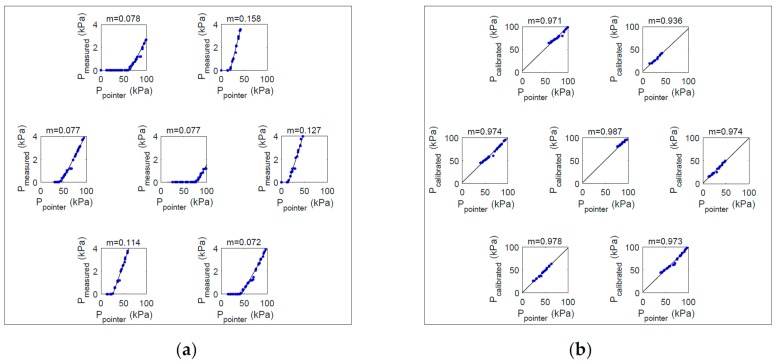
Jig test result of each channel of the sensor: (**a**) output of the seven channels of the sensor module before calibration: (**b**) output of the seven channels of the sensor module after calibration. The inner box shows each channel of the sensor and the graph shows the output (*y*-axis) according to the applied pressure (*x*-axis). Note: m = the slope of the regression equation.

Figure 9 shows results of the jig test for each channel of the sensor module. In Figure 9, the inner box shows each channel of the sensor and the graph shows the output (*y*-axis) according to the applied pressure (*x*-axis). In Figure 9a, the slope of the regression curve between input and output was found to be between 0.077–0.158 and was saturated over a certain interval. Therefore, we carried out the calibration by deriving and applying a conversion equation that transforms the fitting curve generated with the actual measured values into an ideal fitting curve. We did the calibration with the following procedure. First, we created a linear regression model with the values obtained from each sensor. At this time, the generated linear regression model output the fitted pressure by inputting the measured pressure value as $P_{meas-fitted}=a_{1}P_{meas}+b_{1}$. To set the ideal output value, we created a linear regression model for the values generated by the simulation. The generated model was $P_{ideal-fitted}=a_{2}P_{ideal}+b_{2}$. As a correction function, we used a linear function, such as f(x)=mx+n. At this time, in order to find values for the two unknowns, m and n, we derived a system of equations, such as Equation (1), using two random points, $x_{1},\;x_{2}$. Equation (1) can be expressed as Equation (2) using a matrix, and *m* and *n* can be calculated as Equation (3) by matrix operation.

(1) $$\begin{array}{c} mP_{meas-fitted}\left(x_{1}\right)+n=P_{ideal-fitted}\left(x_{1}\right)\\ mP_{meas-fitted}\left(x_{2}\right)+n=P_{ideal-fitted}\left(x_{2}\right) \end{array}$$

(2) $$\left(\begin{array}{cc} m & n \end{array}\right)\left(\begin{array}{cc} P_{meas-fitted}\left(x_{1}\right) & P_{meas-fitted}\left(x_{2}\right)\\ 1 & 1 \end{array}\right)=\left(\begin{array}{cc} P_{ideal-fitted}\left(x_{1}\right) & P_{ideal-fitted}\left(x_{2}\right) \end{array}\right)$$

(3) $$\left(\begin{array}{cc} m & n \end{array}\right)=\left(\begin{array}{cc} P_{ideal-fitted}\left(x_{1}\right) & P_{ideal-fitted}\left(x_{2}\right) \end{array}\right){\left(\begin{array}{cc} P_{meas-fitted}\left(x_{1}\right) & P_{meas-fitted}\left(x_{2}\right)\\ 1 & 1 \end{array}\right)}^{-1}$$

We were, thus, able to calculate the corrected pressure for each channel, as shown in Equation (4).

(4) $$P_{calibrated}=mP_{meas}+n$$

Figure 9b shows the calibrated output of each channel of the sensor module. After calibration, the regression slope for the input–output relationship of the sensor was 0.983–1.000, showing a linear characteristic and saturation in the non-clipping range.

**Figure 10:**
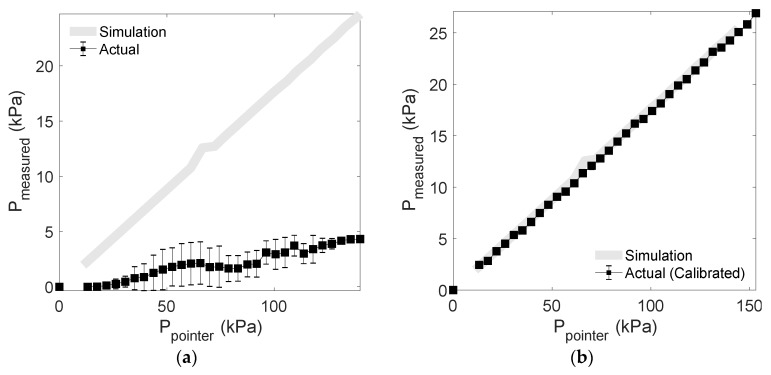
Sensor output: (**a**) averaged sensor output of the seven channels of the sensor module; (**b**) calibrated sensor output of the seven channels of the sensor module.

Figure 10 shows the average pressure and deviation of the output signal after applying pressure to the sensor using the pointer jig. Figure 10a shows the average of the results from reconverting the ADC value to kPa when measured by the channel after applying pressure. As shown in Figure 10a, the full-scale output is different for each channel of the sensor and the thickness of the coated PDMS is different, so each sensor has a deviation, and calibration of the measured value is required for each channel. Figure 10b shows the average and standard deviation of the pressure corrected for each channel by the above equation. In Figure 10b, it can be seen that the accuracy of the measurement result is significantly improved by the correction. In the calibrated output, root mean square error (RMSE) is 0.267 kPa. In the calibration conversion, the saturated measured values, which exhibit no change, are excluded.

### 3.3. Result of Pulse Wave Measurement

**Figure 11:**
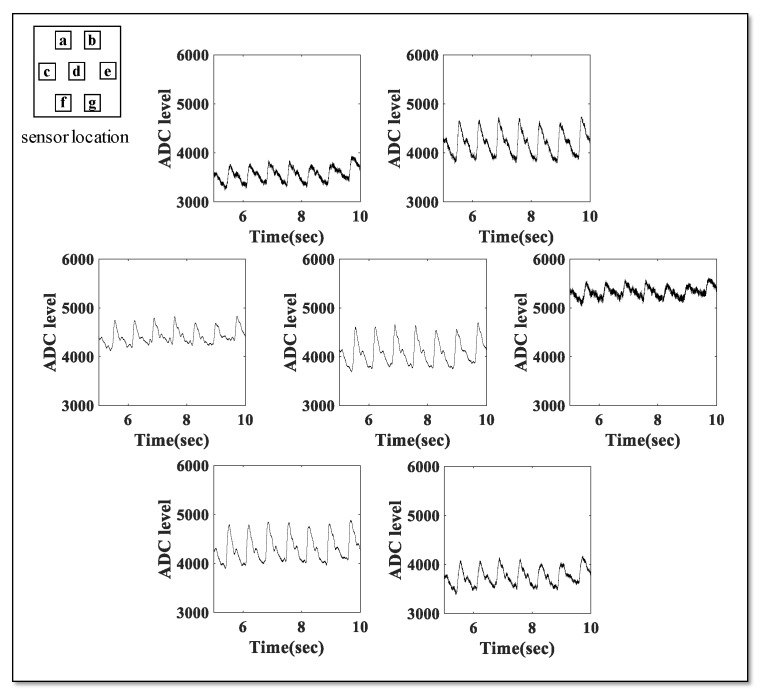
Pulse wave measurement result for each channel.

Figure 11 shows the pulse wave acquired by recording the signal on the radial artery for each channel of the developed pressure sensor. We confirmed that the pulse wave shape is observed in all the sensors, although there are differences in the measured values.

## 4. Discussion

The pulse wave test results show the possibility of using the developed sensor to measure radial arterial pressure propagation according to the cardiac contraction. In particular, the hexagonal sensor arrangement proposed in this study can be used for pulse wave measurement, which is less influenced in the direction of wearing of the sensor module. In addition, the radial artery position is expected to provide a margin of about 10–14 mm corresponding to the width of the sensor unit. However, for the sensor structure, this study did not verify the possibility of acquiring the signal when the arterial position could not be detected accurately, for example, when the sensor was biased in one direction. However, given the results obtained in this study, we can assume that if we can position the sensor on the radial artery with an error of less than 10 mm, we are likely to acquire a signal. In order to observe the sensor response according to the external pressure, we modeled the FEM simulation in the same manner as the actual pressurization environment in order to derive the physio-dynamic simulation results of how the externally applied pressure was delivered to the sensor. The results of the FEM test indicated that externally applied forces were delivered linearly to the sensor. However, attenuation caused by material properties was also observed in the course of the force propagation through PDMS.

Actual pressure test results using a jig showed a linear pressure change similar to the simulation without saturation area. The actual pressurization test and FEM simulation results show that the relationship between the external force and the stress delivered to the sensor surface is linear. However, there is a difference in output of each channel of the developed sensor module. The main cause of this deviation is the full-scale output deviation of the sensor. XGZP2004, a commercial sensor used in sensor module configuration, has a full-scale output of 50–90 mV, so the relative deviation is quite large, which is unavoidable. Other factors were the irregularity in the coating thickness of PDMS. PDMS covers the area surrounded by the dam. At this time, the diluted liquid PDMS was poured and cured. In this process, due to the possibility of process errors such as bubbles occurring, the coating is not completely uniform. Moreover, the minute alignment error may have existed between the jig pointer and the sensor during the jig test. Differences between the channels that result from the sensor fabrication process are inevitable, so a calibration procedure to reduce the differences between the sensors is very important. The linear regression correction suggested in this study effectively reduces the channel-to-channel differences (Figure 9 and Figure 10). Additionally, the difference between the FEM and the actual measurement may be due to the simulation error related to the microstructure. In Chen’s research, it was suggested that the design of microstructures would be unacceptable if single-crystal silicon was postulated as the isotropic material in the FEM simulation [29]. In this study, the microstructure of the sensor was ignored and FEM was set as a solid chip, which may have caused overestimation of the simulation results.

Evaluation of the sensor response to external pressure using the jig showed that the pressure sensor responded when pressurized to a pressure above 30 kPa (~225 mmHg). Hence, a pressure of 30 kPa or more should be applied even when the sensor is located near the radial artery in order to obtain a pulse wave. However, unlike the sensor response evaluation, in which a single sensor is placed on a flat bottom and presses a certain area vertically with the jig, in the radial artery pulse wave measurement, the pulse wave is obtained by pressing the center of the multi-channel pressure sensor PCB substrate on the actual radial artery. In this case, it is difficult to apply the evaluation result of the jig pressure test directly to the wrist pulse wave measurement, because the difference in the total stress and the pressure applied to the sensor, as well as the difference in the total area and pressure being applied, must be considered. In future studies, it is necessary to conduct research to calibrate the pressure value measured by the pressure sensor through the FEM test and the push–pull test on the actual radial artery and wrist models. In addition, research on the minimum pressure needed to acquire pulse waves, a mechanism for uniformly positioning the radial artery sensor, and a group of subjects with wrists of various thicknesses and shapes need to be conducted.

## 5. Conclusions

This study evaluated the characteristics of the newly proposed radial artery pulse wave sensor and the possibility of pulse wave measurement by using FEM simulation, a push–pull pressure test, and a pulse wave measurement experiment. We found that each channel of the developed multi-channel sensor has a deviation, but that the deviation can be minimized by a correction. In addition, the proposed structure might be applicable as a pulse wave sensor with a larger measurement area. Placing the sensor on the radial artery can be accomplished by simply increasing the area of the sensor, however, the results of this study may be used to estimate the radial artery position based on the multi-array structure. However, since the final application of the developed sensor is pulse wave measurement of a human subject, one must verify its robustness against inter-individual differences in anatomical physiology.

## Figures and Tables

**Table 1:** Specifications of pressure sensor (XGZP2004, CFSensor Co., Ltd., Wuhu, China).

Specifications	Value
Size	2.0 × 2.0 × 0.4 mm
Range	0–40 kPa, (0–300 mmHg)
Full scale output	50–90 mV
Repeatability	±0.2% full scale
Non-linearity	±0.3% full scale
Hysteresis	±0.2% full scale
Ambient temperature	−40–125 °C

**Table 2:** Material characteristic for finite element model (FEM) simulation.

Property	Materials			
	PDMS [25]	FR-4 [26]	Sensor (Silicon Substrate) [27]	PLA [28]
Young’s modulus (MPa)	2.05	2.2 × 10^4^	1.65 × 10^5^	3.5 × 10^4^
Poisson’s ratio	0.499	0.18	0.22	0.36
